# Frequency of Hemorrhage on Follow Up Imaging in Stroke Patients Treated With rt-PA Depending on Clinical Course

**DOI:** 10.3389/fneur.2019.00368

**Published:** 2019-04-16

**Authors:** Johannes Schurig, Karl Georg Haeusler, Ulrike Grittner, Christian H. Nolte, Jochen B. Fiebach, Heinrich J. Audebert, Matthias Endres, Andrea Rocco

**Affiliations:** ^1^Center for Stroke Research Berlin, Charité–Universitätsmedizin Berlin, Berlin, Germany; ^2^Department of Neurology, Charité–Universitätsmedizin Berlin, Berlin, Germany; ^3^Department of Neurology, Universitätsklinikum Würzburg, Würzburg, Germany; ^4^Insitute of Biometry and Clinical Epidemiology, Charité – Universitätsmedizin Berlin, Berlin, Germany; ^5^Berlin Institute of Health (BIH), Berlin, Germany; ^6^German Center for Neurodegenerative Diseases, Bonn, Germany

**Keywords:** thrombolysis, stroke, stroke management, magnetic resonance imaging, computerized tomography, intracerebral hemorrhage

## Abstract

**Background:** According to current guidelines, stroke patients treated with rt-PA should undergo brain imaging to exclude intracerebral bleeding 24 h after thrombolysis, before the start of medical secondary prevention. However, the usefulness of routine follow-up imaging with regard to changes in therapeutic management in patients without neurological deterioration is unclear. We hypothesized that follow up brain imaging solely to exclude bleeding in patients who clinically improved after rt-PA application may not be necessary.

**Methods:** Retrospective single-center analysis including stroke patients treated with rt-PA. Records were reviewed for hemorrhagic transformation one day after systemic thrombolysis and brain imaging-based changes in therapeutic management. Twenty-four hour after thrombolysis patients were divided into four groups: (1) increased NIHSS score; (2) unchanged NIHSS score; (3) improved NIHSS score and; (4) NIHSS score = 0.

**Results:** Out of 188 patients (mean age 73 years, 100 female) receiving rt-PA, 32 (17%) had imaging-proven hemorrhagic transformation including 11 (6%) patients with parenchymal hemorrhage. Patients in group (1, 2) more often had hypertension (*p* = 0.015) and more often had parenchymal hemorrhage (9 vs. 4%; *p* < 0.206) compared to group (3, 4) and imaging-based changes in therapeutic management were more frequent (19% vs. 6%; *p* = 0.007). Patients of group (3, 4) had no changes in therapeutic management in 94% of the cases. Patients in group (4) had no hemorrhagic transformation in routine follow-up brain imaging.

**Conclusions:** Frequency of hemorrhagic transformation in Routine follow-up brain imaging and consecutive changes in therapeutic management were different depending on clinical course measured by NHISS score.

## Introduction

Brain imaging 24–36 h after systemic thrombolysis for acute ischemic stroke is recommended in American Stroke Association and European Stroke Organization guidelines (1, 2). Brain imaging is performed to detect secondary bleeding or hemorrhagic transformation in order to adapt medical stroke prevention if necessary. Guideline recommendations are based on the results of the first study on rt-PA (3) demonstrating rt-PA-related bleeding in 11% of all patients.

According to retrospective observational studies, there is evidence for the utility of a follow-up CT scan in stroke patients with clinical signs of deterioration after thrombolysis. However, the benefit of repeated brain imaging is under discussion (4–6). On the one hand, clinicians want to confirm the diagnosis of ischemic stroke, and obtain further information on lesion location, stroke pattern and size before starting medical secondary stroke prevention. On the other hand, in the absence of significant clinical signs, additional imaging places the patients at risk of, potentially unnecessary, treatment delay of antiplatelet therapy, causes an interruption in monitoring and—in case of CT—exposes patients to additional avoidable radiation. Following these arguments, 3 recent retrospective single center studies discussed that routine follow-up CT scan 24 h after thrombolysis may be omitted in selected patients (7–9):

The aim of our study was to point out if routine follow-up brain imaging (CT or MRI) 12–36 h after rt-PA treatment does influence therapeutic management in stroke patients with or without clinical deterioration. Based on clinical experience, we hypothesized that follow-up brain imaging is not needed in stroke patients who clinically improved in the NIHSS score after rt-PA administration.

## Material and Methods

The data supporting the findings of this study are available from the corresponding author upon reasonable request. Hospitalized stroke patients treated with rt-PA in 2015 at the Department of Neurology, Charité–Universitätsmedizin Berlin, Germany were extracted from a local stroke registry. Data were collected following a standard procedure and in accordance with the ethical and privacy policy guidelines of the Charité.

Patients treated with r-tPA for acute stroke who received a head CT or MRI 12–36 h after rt-PA treatment were included. Patients who were treated with intra-arterial therapy and with missing documentation were excluded.

All patients were treated with a rt-PA dosage of 0.9 mg/kg body weight with a maximal dosage of 90 mg rt-PA according to local regulations. As part of standard care, all stroke patients were closely monitored on stroke unit. Vital-parameter and neurological status were monitored and assessed every 6 h or more frequently. Demographic data, past medical history and cardiovascular risk factors (i.e., history of hypertension, hypercholesterolemia, diabetes mellitus and atrial fibrillation) as well as changes in therapeutic management due to routine brain imaging 12–36 h after thrombolysis were retrieved from medical records. Changes in therapeutic management were predefined as follows: (1) subsequent changes or delay in medical secondary stroke prevention (antiplatelet therapy [APT], intravenous or oral anticoagulation [OAC]) for ≥48 h after repeated brain imaging; (2) surgery because of intracerebral bleeding (3) any change in blood pressure management after follow-up brain imaging.

All follow-up brain images were reviewed by a radiologist or neuro-radiologist as part of routine care. Hemorrhagic transformation (HT) was stratified into hemorrhagic infarction (HI) 1 and 2 and parenchymal hematoma (PH) 1 and 2, respectively, in accordance with the European Cooperative Acute Stroke Study II (10). In the case of PH bleeding volume was measured using the ABC/2 method (11).

We divided all patients into 4 groups in accordance to the calculated change in the NIHSS score between admission and 24 h after systemic thrombolysis as follows: (1) increased NIHSS score compared to admission; (2) unchanged NIHSS score; (3) improved NIHSS score and; (4) NIHSS score = 0.

### Statistical Analysis

Dichotomized categorical data were compared using Pearson χ^2^ test or Fisher's exact test where appropriate. Continuous data were compared using Mann–Whitney U test or *t*-Test where appropriate. We calculated positive predictive values (PPV) and 95%CI for changes in therapeutic management.

For continuous or categorical data with more than 2 groups, data were compared using Kruskal–Wallis test or Pearson χ^2^ test where appropriate. All tests were performed at a two-sided significance level of 0.05 level. As this was an exploratory analysis, no adjustments were made for multiple comparisons. All tests were performed via SPSS Version 23 (12). The 95% confidence intervals for positive predictive values, sensitivity and specificity were calculated with STATA Version 14 (13).

## Results

### Baseline Characteristics

We included 226 patients with suspected stroke. Thirty-four patients who were treated with intra-arterial therapy, 3 patients with incomplete documentation and 1 patient with death due to cardiac arrest before follow up brain imaging were excluded, leaving overall, 188 patients for analysis. In 9 patients the diagnosis of a stroke mimic was made in the latter course of disease including follow up brain imaging. Baseline characteristics of these patients are shown in Table 1. Eighteen out of 188 patients (10%) had a deterioration according to NIHSS score within 24 h after systemic thrombolysis (group 1), 60 patients (32%) had no change in the NIHSS score (group 2), 82 (44%) had improvement of the in NIHSS score (group 3), and 28 patients (15%) had an NIHSS score equal to 0 (group 4).

**Table 1:** Baseline characteristics of the study cohort and all subgroups.

	**Study cohort (*n* = 188)**	NIHSS deterioration* (*n* = 18)	No change in NIHSS* (*n* = 60)	NIHSS improvement* (*n* = 82)	NIHSS = 0* (*n* = 28)	***P*-Value *KW*-Test^**a**^*/χ*^**2**^-Pearson^**b**^**
**LABORATORY DIAGNOSTICS (BLOOD) AND CLINICAL PARAMETERS**						
Age, mean [SD]	73.3 [13.8]	78.3 [13.5]	73.6 [12.7]	74.2 [13.6]	66.9 [15.4]	0.066^a^
Male sex, % (*n*)	46.8 (88)	27.8 (5)	50.0 (30)	48.8 (40)	46.4 (13)	0.393^b^
MRI after 24h, % (*n*)	65.4 (123)	33.3 (6)	63.3 (38)	67.1 (55)	85.7 (24)	0.004^*b*^
NIHSS on admission, median points [IQR]	6[3-13]	9[5-17]	4[2-12]	8[5-16]	3[2-5]	< 0.001^a^
eGFR in ml/min/1.73 m^2^, median [IQR]	68 [50.5–84.5]	58 [54.5–78.5]	72 [47.75–86.75]	62 [50–84]	77 [54–84]	0.572^a^
HbA1c in %, median [IQR]	5.8 [5.4–6.3]	5.9 [5.4–6.1]	5.8 [5.4–6.2]	5.8 [5.4–6.7]	5.6 [5.1–6.0]	0.311^a^
SBP at admission in mmHg, median [IQR]	150 [130–170]	148 [129–172]	160 [130–176]	150 [130–170]	150 [130–178]	0.944^a^
First SBP after thrombolysis in mmHg, median [IQR]	150 [134–166]	165 [145–187]	156 [136–172]	145 [131–162]	149 [130–170]	0.035^a^
BS at admission in mmol/l, median [IQR]	6.8 [5.9–8.7]	6.6 [6.2–9.8]	7.0 [6.4–8.8]	6.7 [5.8–8.4]	6.6 [5.8–8.0]	0.580^a^
First BS after thrombolysis in mg/dl, median [IQR]	6.7 [5.6–8.1]	6.6[5.6–7.7]	7.2 [5.8–8.4]	6.7 [5.6–8.3]	6.4 [5.5–7.8]	0.634^a^
**CARDIOVASCULAR RISK FACTORS**						
DM, % (*n*)	25 (46)	38.9 (7)	26.3 (15)	25.6 (21)	11.1 (3)	0.195^b^
AHT, % (*n*)	78.3 (144)	88.2 (15)	75.4 (43)	85.4 (70)	57.1 (16)	0.012^b^
HLP, % (*n*)	53.2 (99)	47.1 (8)	55.9 (33)	51.2 (42)	57.1 (16)	0.864^b^
Previous IS or TIA, % (*n*)	24.4 (44)	31.3 (5)	31.6 (18)	16 (13)	30.8 (8)	0.131^b^
CAD % (*n*)	13.7 (25)	12.5 (2)	15.5 (9)	11.1 (9)	18.5 (5)	0.761^b^
AFib % (n)	19.1 (35)	23.5 (4)	19.3 (11)	23.2 (19)	3.7 (1)	0.155^b^
PAOD, % (*n*)	5 (9)	0 (0)	5.3 (3)	6.2 (5)	3.7 (1)	0.756^b^
CHF, % (*n*)	9.4 (17)	12.5 (2)	10.5 (6)	10 (8)	3.7 (1)	0.724^b^
Nicotin abuse, % (*n*)	17.4 (32)	22.2 (4)	21.1 (12)	14.6 (12)	14.8 (4)	0.710^b^

Results are presented as p-values using Pearson Chi squared ^(b)^ or Kruskal-Wallis test ^(a)^ when appropriate. *24 h after systemic rt-PA application; AFib indicates atrial fibrillation; BS, blood sugar; CAD, coronary artery disease; CHF, congestive heart failure; DM, diabetes mellitus; eGFR, estimated glomerular filtration rate; HLP, hyper lipoproteinemia; AHT, arterial hypertension; IS, ischemic stroke; INR, international normalized ratio; IQR, interquartile range; NIHSS, National Institutes of Health Stroke Scale; PAOD, peripheral arterial occlusive disease; TIA, transient ischemic attack; SBP, systolic blood pressure.

We found differences in baseline characteristics for the four different groups with regard to prevalence of arterial hypertension (lowest in group 4), median admission NIHSS score (lowest in group 4). Additionally, those with NIHSS score of 0 were on average younger (mean age 67) compared to other groups (mean age 74), had less often atrial fibrillation (4% vs. 19% or more), and less often diabetes mellitus (11% vs. 26% or more). Results are shown in Table 1.

### Comparing Stroke Patients With and Without Clinical Improvement in NIHSS Score After Thrombolysis

Comparing group 1–2 to group 3–4, differences in baseline demographics or cardiovascular risk factors were not statistically significant.

Patients in group 3–4 more often underwent follow-up MRI (72% vs. 56% in group 1–2, *p* = 0.031) and had lower systolic blood pressure values measured directly after the beginning of thrombolysis (median: 147 mmHg vs. 159 mmHg in group 1–2, *p* = 0.015).

### Comparing Stroke Patients With and Without Clinical Deterioration in NIHSS Score After Thrombolysis

Patients in group 2–4 more often underwent follow-up MRI (69% vs. 33% in group 1, *p* = 0.003) and had lower systolic blood pressure values measured directly after the beginning of thrombolysis (median: 150 mmHg vs. 165 mmHg in group 1, *p* = 0.020).

### Location of Ischemic Stroke and HT Type in Follow-Up Brain Imaging

We found differences in imaging findings in follow-up brain imaging 12–36 h after thrombolysis for the four different groups with regard to location of ischemic stroke in the anterior and medial cerebral artery territory (lowest in group 4), no ischemic stroke in follow-up brain imaging (highest in group 4), HI2 (highest in group 2), PH2 (highest in group 1). Comparing group 1–2 to group 3–4, HI2 (10% vs. 1%, *p* = 0.004) and PH2 (8% vs. 2%, *p* = 0.049) were found more often in group 1–2 and there was a trend toward a higher volume of PH in group 1–2 missing statistically significance (median volume: 29.5 ml vs. 1.5 ml, *p* = 0.059). Comparing group 1–2 to group 3–4, differences in location of ischemic stroke were not statistically significant (Table 4).

### Incidence of Other Intracranial Bleeding Types

In addition to PH on follow-up brain imaging 12–36 h after thrombolysis, there were 3 patients suffering from concomitant intraventricular hemorrhage (IVH) and 2 patients sustaining concomitant subarachnoid hemorrhage (SAH). One of three patients sustaining IVH had a neurological deterioration evident in NIHSS scoring and the other 2 patients remained clinically stable. Both patients with concomitant SAH deteriorated within 24 h after IVT application.

In one case, the diagnosis of amyloid angiopathy was newly made on the basis of multiple microbleeds seen on MRI on admission. This patient neurologically improved as captured on the NIHSS within 24 h but follow-up imaging revealed a PH2 (volume 2.1 ml).

### Impact of HT on Changes in Therapeutic Management

Patients in group 2–4 had significantly less often changes in therapeutic management compared to group 1 (10% vs. 28%; *p* = 0.026, Table 2), The positive predictive value (PPV) for no changes in therapeutic management was 90% (95%CI: 84.5–94.1%) with a sensitivity of 92% (95%CI: 87.0–95.8%) and a specificity of 23% (95%CI: 7.8–45.4%).

Table 2Imaging findings and changes in therapeutic management.

**Table:** 

	NIHSS deterioration* (*n* = 18)	No change in NIHSS* (*n* = 60)	NIHSS improvement* (*n* = 82)	NIHSS = 0* (*n* = 28)	***P-Value χ*^**2**^*Pearson***
**IMAGING FINDINGS**					
No HT	12 (66.6%)	42 (70%)	69 (84.1%)	28 (100%)	0.006
HI	1 (5.5%)	13 (21.7%)	7 (8.5%)	0 (0%)	0.010
PH	5 (27.8%)	2 (3.3%)	4 (4.9%)	0 (0%)	< 0.001
**CHANGES IN THERAPEUTIC MANAGEMENT**					
Delayed med due to HT in CI	5 (27.8%)	4 (6.7%)	2 (2.4%)	0 (0%)	0.000
Any change in BP management due to HT in CI	4 (22.2%)	8 (13.3%)	6 (7.3%)	0 (0%)	0.051
Changes in therapeutic management due to HT in CI	5 (27.8%)	10 (16.7 %)	7 (8.5%)	0 (0%)	0.015

**Table:** 

	NIHSS deterioration or no change* **(*****n*** **=** **78)**	**NIHSS improvement or NIHSS** **=** 0* **(*****n*** **=** **110)**	
**IMAGING FINDINGS**			
No HT	54 (69.2%)	97 (88.2%)	0.002
HI	14 (17.9%)	7 (6.4%)	0.013
PH	7 (9.0%)	4 (3.6%)	0.0124
**CHANGES IN THERAPEUTIC MANAGEMENT**			
Delayed med due to HT in FU-BI	9 (11.5%)	2 (1.8%)	0.005
Any change in BP management due to HT in FU-BI	12 (15.4%)	6 (5.4%)	0.023
Changes in therapeutic management due to HT in FU-BI	15 (19.2%)	7 (6.4%)	0.007
	NIHSS deterioration* **(*****n*** **=** **18)**	**No Change in NIHSS, NIHSS improvement or NIHSS** **=** 0* **(*****n*** **=** **170)**	
**IMAGING FINDINGS**			
No HT	12 (66.6%)	144 (84.7%)	0.053
HI	1 (5.5%)	20 (11.8%)	0.426
PH	5 (27.8%)	6 (3.5%)	< 0.001
**CHANGES IN THERAPEUTIC MANAGEMENT**			
Delayed med due to HT in FU-BI	5 (27.8%)	6 (3.6%)	< 0.001
Any change in BP management due to HT in FU-BI	4 (22.2%)	14 (8.2%)	0.055
Changes in therapeutic management due to HT in FU-BI	5 (27.8%)	17 (10%)	0.026
	NIHSS deterioration, no change in NIHSS or NIHSS improvement* **(*****n*** **=** **160)**	**NIHSS** **=** 0* **(*****n*** **=** **28)**	
**IMAGING FINDINGS**			
No HT	128 (80%)	28 (100%)	0.009
HI	21 (13.1%)	0 (0%)	0.042
PH	11 (6.9%)	0 (0%)	0.153
**CHANGES IN THERAPEUTIC MANAGEMENT**			
Delayed med due to HT in FU-BI	11 (6.9%)	0 (0%)	0.153
Any change in BP management due to HT in FU-BI	18 (11.3%)	0 (0%)	0.062
Changes in therapeutic management due to HT in FU-BI	22 (13.8%)	0 (0%)	0.037

The results of dichotomiced data regarding follow-up brain imaging findings for hemorrhagic transformation and management changes are given in this table. Results are presented as p-values using Pearson Chi squared or Fisher exact test when appropriate. There was no patient with cranial surgery due to hemorrhagic transformation in follow-up brain imaging. ^*^24 h after systemic rt-PA application; BP indicates blood pressure; Delayed Med, delayed medication for more than 48 h after intravenous thrombolysis (anti-platelet therapy or anticoagulation); FU-BI, routine follow-up brain imaging after 12–36 h; HI, Hemorrhagic infarction; HT, hemorrhagic transformation; NIHSS, National Institutes of Health Stroke Scale; PH, Parenchymal hematoma.

Patients in group 3–4 had even less often changes in therapeutic management compared to patients in group 1–2 (6% vs. 19%; *p* = 0.007, Table 2). The PPV for no changes in therapeutic management was 94% (95%CI: 87.3–97.4%, Table 3) with a sensitivity of 62% (95%CI: 54.2–69.5%, Table 3) and a specificity of 68% (95%CI: 45.1–86.1%, Table 3).

**Table 3:** Sensitivity, specificity, positive and negative predictive value of improvement in NIHSS for imaging findings and changes in therapeutic management.

	**Sensitivity in % (CI-95%)**	**Specificity in % (CI-95%)**	**PPV in % (CI-95%)**
HT^*^	63.4 (55.4–71.0)	65.6 (46.8–81.4)	90.0 (82.8–94.9)
PH^*^	59.9 (52.4–67.2)	63.6 (30.8–89.1)	96.3 (91.0–99.0)
HI^*^	61.7 (53.8–69.1)	66.7 (53.8–69.1)	93.6 (82.8–94.9)
Changes in therapeutic management due to HT^*^	62.0 (54.2–69.5)	68.2 (45.1–86.1)	93.6 (87.3–97.4)

*The sensitivity, specificity and positive predictive value of improvement in NIHSS [Group (3, 4)] for imaging findings and changes in therapeutic management due to hemorrhagic transformation in routine follow-up brain imaging after 12–36 h are given in this table. Results are presented in percentages using STATA 14 for determination of confidence intervals*.

* *in routine follow-up brain imaging after 12–36 h. CI indicates confidence interval; HI, hemorrhagic infarction; HT, hemorrhagic transformation; NIHSS, National Institutes of Health Stroke Scale; NPV, negative predictive value; PH, parenchymal hematoma; PPV, positive predictive value*.

Table 4Imaging findings and stroke location due to routine follow-up brain imaging after 12–36 h.

**Table:** 

**Imaging findings**	NIHSS deterioration* (*n* = 18)	No change in NIHSS* (*n* = 60)	NIHSS improvement* (*n* = 82)	NIHSS = 0* (*n* = 28)	***P*-Value χ^**2**^ Pearson^**a**^*KW*-Test^**b**^*MWU*-Test^**c**^**
IS in ACA or MCA territory	14 (77.8%)	36 (60.0%)	55 (67.1%)	8 (28.6%)	0.001^a^
IS in vertebrobasilar territory	3 (16.7%)	18 (30.0%)	28 (34.1%)	6 (21.4%)	0.366^a^
No IS in FU-BI	1 (5.6%)	10 (16.7%)	8 (9.8%)	16 (57.1%)	< 0.001^a^
HI 1	1 (5.6%)	5 (8.3%)	6 (7.3%)	0 (0%)	0.488^a^
HI 2	0 (0%)	8 (13.3%)	1 (1.2%)	0 (0%)	0.003^a^
PH 1	1 (5.6%)	0 (0%)	2 (2.4%)	0 (0%)	0.309^a^
PH 2	4 (22.2%)	2 (3.3%)	2 (2.4%)	0 (0%)	0.001^a^
Volume of PH in ml, median [IQR]	87.9 [15.1–112.1]	14.2 [5.6–n.a.]	1.5 [0.7–9.6]	n.a.	0.129^b^

**Table:** 

**Imaging findings**	NIHSS deterioration or no change* **(*****n*** **=** **78)**	**NIHSS improvement or NIHSS** **=** 0* **(*****n*** **=** **110)**	
IS in ACA or MCA territory	50 (64.1%)	63 (57.3%)	0.346^a^
IS in vertebrobasilar territory	21 (26.9%)	34 (30.9%)	0.554^a^
No IS in FU-BI	11 (14.1%)	24 (21.8%)	0.181^a^
HI 1	6 (7.7%)	6 (5.5%)	0.536^a^
HI 2	8 (10.3%)	1 (0.9%)	0.003^a^
PH 1	1 (1.3%)	2 (1.8%)	0.773^a^
PH 2	6 (7.7%)	2 (1.8%)	0.049^a^
Volume of PH in ml, median [IQR]	29.5 [5.6–95.4]	1.5 [0.6–13.4]	0.059^c^

Results are presented as p-values using χ^2^ Pearson ^(a)^, Kruskal-Wallis ^(b)^ or Mann–Whitney U test ^(c)^ when appropriate. ^*^24 h after systemic rt-PA application; ACA, anterior cerebral artery; FU-BI, routine follow-up brain imaging after 12–36 h; HI, Hemorrhagic infarction; IS, ischemic stroke; MCA, medial cerebral artery; NIHSS, National Institutes of Health Stroke Scale; PH, Parenchymal hematoma.

Patients in group 4 had no HT and hence significantly less often management changes compared to patients in group 1–3 (0% vs. 14%; *p* = 0.037, Table 2). The PPV for no changes in therapeutic management was 100% (95%CI: 87.7–100%) with a sensitivity of 17% (95%CI: 11.5–23.4%) and a specificity of 100% (95%CI: 84.6–100%).

In a sub analysis excluding the 9 patients who were diagnosed as stroke mimics in the latter course of the hospital stay, the PPV for no changes in therapeutic management in group 1 vs. group 2–4 was 89% (28% vs. 11% *p* = 0.035), in group 1–2 vs. group 3–4 93% (20% vs.7% *p* = 0.008) and in group 1–3 vs. group 4 100% (14% vs. 0% *p* = 0.061).

## Discussion

Diagnosis and treatment decisions in stroke require brain imaging. Following treatment with rt-PA, guidelines recommend to repeat brain imaging 24–36 h after application.

The reasons to conduct imaging after acute stroke treatment are multiple; only one of them is to understand hemorrhagic evolution. Others include understanding mass effect, considering the need for hemicraniectomy or osmotic therapy, understanding prognosis, planning rehabilitation and confirm diagnosis. In case of clinical worsening during or after thrombolysis immediate brain imaging is performed in clinical practice specially to exclude a hemorrhage. In patients who tolerated rt-PA well and demonstrated improvement, the question remains, whether this obligatory follow-up brain-imaging is necessary before starting secondary prevention treatment.

Comparing present literature (7–9), our study has a similar sample size compared to other reports, but answers more specifically clinically relevant aspects:

Dharamasaroja et al were the first who questioned the need for a follow-up CT after rt-PA treatment in patients with early clinical/neurological improvement, based on a retrospective study including 200 stroke patients. However, they did not mention any clinical impact (7). Furthermore, George et al. reported similar results as our study, based on a retrospective analysis including 200 patients, but did not describe the changes in therapeutic management in the subgroups who had findings of bleeding on imaging (8). Moreover, Guhwe et al. reported that clinicians changed the therapeutic management following routine CT scans in just one out of 136 clinically stable stroke patients. However, they reported solely alterations in blood pressure management as a change in therapeutic management after HT in follow-up brain imaging. No other changes in management were documented (9). In a recently published abstract, Sevilis et al. analyzed 280 stroke patients after rt-PA and showed in 5% a delay in initiation of antithrombotic medication for asymptomatic patients due to follow-up brain imaging. However, the data has not been published (14) yet. To date, our study is the only one that considered several aspects of changes in therapeutic management, such as delay in antiplatelet agents or oral anticoagulation, blood pressure management, or neurosurgical interventions in relation to HT after thrombolytic treatment.

Our cohort showed more frequent changes in therapeutic management of asymptomatic patients due to HT in follow-up brain imaging (10%) compared to Guhwe et al. (1 out of 136 patients) and Sevilis et al. (5% of delay in antithrombotic medication, unpublished data).

In our cohort, the number of overall HT in follow-up brain imaging in the case of clinical improvement in the NIHSS score after 24 h was 10% with no cases of other bleeding types such as IVH and SAH. This information leaded in 6% of this group to an overall change in therapeutic management and in 2% to a delay in the start of medical secondary prevention. In the case of complete recovery in the NIHSS score after 24 h no HT occurred in follow-up brain Imaging.

As already mentioned, a control image is not only used to exclude bleeding, but this would be one of the most important reasons for delaying secondary prevention therapy. After thrombolytic treatment in case of hemorrhage, mass effect by malignant cerebral infract or other pathologies, a clinical improvement is unlikely to be expected.

Our data imply that the use of routine follow up brain imaging could be avoided in the case of complete clinical recovery in NIHSS, avoiding, in the case of CT, radiation exposure and above all costs.

However, with 6% of overall changes in therapeutic management due to HT in the case of clinical improvement in NIHSS score, prospective studies are needed to further investigate the impact of these management changes on therapeutic outcome before conclusions could be drawn.

This study has several limitations: (a) data were assessed retrospectively in a single tertiary care University hospital. Although patient care was based on European and National guidelines, variations in clinical practice (e.g., availability of MRI) exist and the results may not be generalizable to other care settings; (b) the number of patients in our cohort is limited in certain subgroups and hence, prospective studies are needed to validate our findings; (c) the percentage of patients undergoing MRI varied across subgroups; (d)despite its proven validity the NIHSS score has certain limitations and may fail to assess clinical alterations in patients with posterior circulation stroke (15); (e) the proportion of patients with mild strokes in our study was high with a median NIHSS of 6 and our results may not be generalized to patients with more severe strokes who frequently persist with important deficits despite improvement.

## Summary

In patients with complete recovery in NIHSS score within 24 h after thrombolysis, routine follow up brain imaging seems not to be mandatory before starting secondary prevention.

## Ethics Statement

All subjects gave written informed consent in accordance with the Declaration of Helsinki. This single-center retrospective analysis received approval from the Ethics Committee of the Charité–Universitätsmedizin Berlin, Germany.

## Author Contributions

JS has made substantial contributions to data acquisition, analysis and interpretation of data and drafted the manuscript. KH has made substantial contributions to conception and design, analysis, and interpretation of data and revised the manuscript critically for important intellectual content. CN and JF made substantial contributions to data acquisition and revised the manuscript critically for important intellectual content. UG has made substantial contributions to analysis and interpretation of data and revised the manuscript critically for important intellectual content. HA and ME revised the manuscript critically for important intellectual content. AR has made substantial contributions to conception and design, analysis and interpretation of data and drafted the manuscript.

### Conflict of Interest Statement

KH reports study grants by Bayer and Sanofi-Aventis, lecture fees/advisory board fees from Bayer, Sanofi-Aventis, Pfizer, Bristol-Myers-Squibb, Boehringer Ingelheim, Daiichi Sankyo, Biotronik, LA Gore, EIP Pharma, and Medtronic. AR received lecture honoraria from Ever Pharma and Bayer HealthCare, as well as congress participation costs from Bayer HealthCare and Bristol- Meyers Squibb Pfizer. ME reports grant support and/or fees paid to the Charité from Bayer, BI, BMS/Pfizer, Daiichi Sankyo, Amgen, Sanofi, Covidien GSK, Ever, Novartis, all outside the submitted work. HA has received speaker or consultancy honoraria from Boehringer Ingelheim, Bayer Healthcare, Sanofi, Daiichi-Sankyo, Pfizer, Bristol-Myers Squibb, Siemens and Novo Nordisk. JF has received consulting, lecture, and advisory board fees from BioClinica, Cerevast, Artemida, Brainomix, and Lundbeck as well as a grant from the German Federal Ministry of Education and Research (01EO0801 and 01EO01301). As PI he receives funding from the European Union Seventh Framework Program [FP7/2007–2013] under grant agreement no. 278276 (WAKE-UP). JF is holding European Patent No 17179320.01-1906. CN received lecture and/or consulting honoraria from Sanofi-Aventis, Pfizer, Bristol-Myers Squibb, Boehringer Ingelheim, and Gore and Associates. The remaining authors declare that the research was conducted in the absence of any commercial or financial relationships that could be construed as a potential conflict of interest.

## References

[1] Powers WJ Rabinstein AA Ackerson T Adeoye OM Bambakidis NC Becker K. 2018 guidelines for the early management of patients with acute ischemic stroke: a guideline for healthcare professionals from the american heart association/american stroke association. Stroke. (2018) 49:e46–110. 10.1161/STR.0000000000000158 29367334

[2] European Stroke Organisation Executive C, Committee ESOW Guidelines for management of ischaemic stroke and transient ischaemic attack 2008. Cerebrovasc Dis. (2008) 25:457–507. 10.1159/000131083 18477843

[3] National Institute of Neurological D, Stroke rt PASSG Tissue plasminogen activator for acute ischemic stroke. N Engl J Med. (1995) 333:1581–7. 7477192 10.1056/NEJM199512143332401

[4] Ertl-Wagner B Brandt T Seifart C Forsting M. Diagnostic and therapeutic consequences of repeat brain imaging and follow-up vascular imaging in stroke patients. AJNR Am J Neuroradiol. (1999) 20:37–42. 9974056

[5] Schneider LB Libman RB Kanner R. Utility of repeat brain imaging in stroke. AJNR Am J Neuroradiol. (1996) 17:1259–63. 8871709 PMC8338518

[6] Collins TR News from the aan annual meeting: routine ct scans after tpa do not effect clinical management, study finds. Neurol Today. (2016) 16:35–6. 10.1097/01.NT.0000483058.68552.99

[7] Dharmasaroja PA Muengtaweepongsa S Dharmasaroja P Post rtpa ct brain may not be mandatory in all stroke patients when resources are limited. Clin Neurol Neurosurg. (2013) 115:285–8. 10.1016/j.clineuro.2012.05.040 22749008

[8] George AJ Boehme AK Dunn CR Beasley T Siegler JE Albright KC. Trimming the fat in acute ischemic stroke: an assessment of 24-h ct scans in tpa patients. Int J Stroke. (2015) 10:37–41. 10.1111/ijs.12293 24894300 PMC4602409

[9] Guhwe M Utley-Smith Q Blessing R Goldstein LB Routine 24-hour computed tomography brain scan is not useful in stable patients post intravenous tissue plasminogen activator. J Stroke Cerebrovasc Dis. (2016) 25:540–2. 10.1016/j.jstrokecerebrovasdis.2015.11.006 26679069

[10] Hacke W Kaste M Fieschi C von Kummer R Davalos A Meier D. Randomised double-blind placebo-controlled trial of thrombolytic therapy with intravenous alteplase in acute ischaemic stroke (ecass ii). Second european-australasian acute stroke study investigators. Lancet. (1998) 352:1245–51. 9788453 10.1016/s0140-6736(98)08020-9

[11] Kothari RU Brott T Broderick JP Barsan WG Sauerbeck LR Zuccarello M. The ABCs of measuring intracerebral hemorrhage volumes. Stroke. (1996) 27:1304–5. 8711791 10.1161/01.str.27.8.1304

[12] IBM Corp IBM SPSS Statistics for Windows, Version 23.0. Armonk, NY: IBM Corp (2015).

[13] StataCorp. Stata Statistical Software: Release 14. College Station, TX: StataCorp LP (2015).

[14] Sevilis T Morantes Gomez L Shah N Wang M Huang D Powers W The effect of 24 hour post-intravenous alteplase ct head on management decisions: a single center experience (s47.004). Neurology. (2016) 86. Available online at: https://n.neurology.org/content/86/16_Supplement/S47.004

[15] Meyer BC Lyden PD. The modified national institutes of health stroke scale: its time has come. Int J Stroke. (2009) 4:267–73. 10.1111/j.1747-4949.2009.00294.x 19689755 PMC2729912

